# Gold Particle Analyser: Detection and quantitative assessment of electron microscopy gold probes

**DOI:** 10.1371/journal.pone.0288811

**Published:** 2023-07-28

**Authors:** Thomas Burgoyne, Clare E. Futter

**Affiliations:** 1 UCL Institute of Ophthalmology, London, United Kingdom; 2 Royal Brompton Hospital, Guy’s and St Thomas’ NHS Foundation Trust, London, United Kingdom; Justus Liebig Universitat Giessen, GERMANY

## Abstract

Gold particle probes are an essential electron microscopy tool to examine protein localisation, as well as protein trafficking. They can be introduced into living cells when conjugated to a protein that is endocytosed or to an antibody against a cell surface protein. Alternatively, gold particles can be introduced into fixed cells or tissue when conjugated to antibodies, immunoglobulin binding molecules or chemical probes applied to permeabilised samples or electron microscopy sections. Colloidal gold particles that have not been enlarged through chemical (gold or silver) enhancement are typically spherical and can be prepared in a range of specific sizes, allowing multiple proteins to be localised within a single sample. The typically homogeneous shape and size of the colloidal gold makes them ideal for computer assisted detection and analysis. Here we demonstrate a program developed to automatically identify two sizes of gold particle and perform a range of analyses that includes (i) distribution and cluster analysis; (ii) selection and analysis of gold particles allocated close to or either side of a membrane; (iii) measurement of organelle size; (iv) estimation of the number of gold particles within an aggregate and (v) the detection of chemically enhanced irregular sized and shaped gold particles. We show this easy-to-use program can greatly assist electron microscopists, to reliably and efficiently analyse gold particles within their images.

## Introduction

Colloidal gold particles are commonly used in transmission electron microscopy (TEM) as probes to determine the localisation of proteins or identify organelles at an ultrastructural level, as well as to provide key knowledge of pathways involved in protein and membrane trafficking. Manual counting of gold particles has been successfully used in many studies to establish the localisation of labelling within cells [1, 2]. Manual analysis can be hampered however by the scarcity or high abundance of gold particles, the potential distance of the gold particle from the antigen and uneven gold enhancement. Automated detection and analysis of labelling, provides a more objective approach that is advantageous when there is a high density of gold particles, or complex analysis is required. This includes analysing gold a set distance from a membrane or structure and grouping gold into clusters, which is not straightforward when using manual approaches. Automated programs have been developed to analyse gold labelling that include automatic detection of gold particles using image thresholding and feature detection algorithms as well as machine learning [3–5]. These show good capability of automatically identifying gold but reported difficulties with clustered gold and detecting enhanced gold labelling. We have therefore developed a program to provide a rapid, unbiased way to determine the number, nearest neighbour distance and clustering of up to two sizes of gold particles. The program also has features to separate gold particles either side of a membrane or given structure, assess aggregated gold and detect gold particles that vary in size and morphology. Here we test this programme using four examples of different types of gold labelling which we have previously analysed by eye:

The first three examples involve immunogold labelling for TEM. This can be performed either by embedding, sectioning and then immunogold labelling of cell or tissue sections (post-embedding labelling) or by immunogold labelling of permeabilised cells or tissues, followed by embedding and sectioning (pre-embedding labelling). Immuno-gold labelling of thawed cryo-sections, often referred to as the Tokuyasu technique after its pioneer [6], requires technically demanding cyro-ultramicrotomy of cells or tissue samples. Antibodies applied directly to thawed sections are followed by incubation with either gold conjugated to a secondary antibody or to the immunoglobulin binding molecules, protein-A or protein-G (proteins isolated from the *Staphylococcus aureus* and *Streptococcal* bacteria respectively that have a high affinity to Fc portion of IgG’s) [7, 8]. Due to the size of the antibodies and antibody binding molecules used, the gold can be up to ~15 to 30 nm away from the antibody binding epitope [9]. To perform pre-embedding labelling on fixed cells or tissue the plasma membrane and possibly intracellular organelles must be permeabilised to allow access to cytoplasmic and intraorganellar epitopes with consequent loss of preservation [10]. Colloidal nanogold particles (<1.5 nm) attached to an antibody Fab fragment or chemical probe (such as streptavidin) are frequently used as they are small enough to enter though gaps in cell membranes created through permeabilization, unhindered [11].

In our first example we have used immunogold labelling of thawed cryosections because it requires access to the lumen of an intracellular organelle. Lysosomal targeting of membrane proteins occurs through their sorting onto the intraluminal vesicles (ILVs) that accumulate within the lumen of endosomes. To monitor sorting of membrane proteins onto ILVs requires access to epitopes within the endosomal lumen and determination of gold proximity to the membranes of the ILV versus the MVB perimeter. Here we use Gold Particle Analyser to compare the ILV sorting of two Ocular Albinism Type 1 (OA1) variants that we previously showed differ in their sorting onto ILVs, taking advantage of the feature to separate gold particles on either side of a membrane [12].

Gold particles of different sizes can be used to detect different antigens on the same thawed cryosections. In our second example we have performed cryo-immunoEM using antibodies to two different rhodopsin epitopes that allow the processing of phagocytosed photoreceptor outer segments to be monitored in retinal pigment epithelial cells. We have previously shown that the rhodopsin C terminus is lost prior to phagosome:lysosome fusion but the N terminus is not lost until lysosomal delivery [13]. Simultaneous monitoring of the gold number, clustering and nearest neighbour of these two epitopes with Gold Particle Analyser provides a readout of phagosome maturation and lysosome fusion.

Our third example involves pre-embedding labelling, which is potentially a more accessible technique than cryo-immunoEM, can be used on cryostat sections and can have improved labelling efficiency, compared with labelling of cryosections. As long as the antigen is not lost during permeabilization, the small size of the gold particle reduces the possibility of antigen masking and there is potentially more antigen available (labelling is not restricted to the surface of the section) [14, 15]. The uneven size and shape of the enhanced gold particles however render quantification potentially challenging. Here we show that Gold Particle Analyser can detect and quantify enhanced nano gold that we have previously used to identify peroxisomes [10], an organelle that has limited morphological features to identify it.

Our final example uses colloidal gold conjugated to BSA that is taken up by living cells via the endocytic pathway and, ultimately, delivered to the lysosome. Cells are then fixed, embedded and sectioned for TEM. We have previously used gold aggregation as a useful readout of lysosomal delivery because when the BSA that stabilises the gold is degraded in the lysosome the gold particles aggregate in the acidic lysosomal lumen [12]. However, when gold is aggregated it becomes difficult to quantify. Here we show that Gold Particle Analyser can quantify gold particles aggregated within lysosomes.

## Materials and methods

### Mouse eyes

The mouse eyes used in the study were existing TEM and cryostat embedded samples from wild type C57Bl6 untreated mice that had been sacrificed by cervical dislocation at a Schedule 1-approved designated establishment in accordance with Animals (Scientific Procedures) Act 1986 (United Kingdom) and Home Office (United Kingdom) guidance rules, adhering to the Association for Research in Vision and Ophthalmology Statement for the Use of Animals in Ophthalmic and Vision Research.

### Immuno-electron microscopy of thawed cryo sections

Mouse eyes and HeLa cells were prepared for immunogold labelling of thawed cryo sections. Mouse eyes were acquired 1 and 2.5 hrs after light onset and fixed in 4% PFA and 0.1% glutaraldehyde in 0.1 M phosphate buffer. HeLa cells were transfected with Myc tagged OA1 or OA1-Δ18 constructs as previously described [12]. Lipofectamine 2000 reagent (Invitrogen) was used following the manufacturer’s guidelines, for 48 hours with the constructs. These samples were fixed in 4% paraformaldehyde in 0.1 M phosphate buffer at pH 7.4. Both the eyes and cell samples were embedded in 12% gelatin and left in 2.3M sucrose overnight at 4°C. The samples were mounted and sectioned using a cryo-ultramicrotome before immunolabelling the sections with antibodies against rhodopsin epitopes 1D4 (Abcam) and RET-P1 (Abcam) as well as Myc (Millipore). Antibodies were detected using rabbit anti-mouse bridging antibody and protein A conjugated to 10 and 15 nm gold (PAG) (UMC Utrecht) following previously described methods [16].

### Pre-embedding labelling

A mouse eye was prepared and pre-embedding labelled performed using an antibody against the 70-kDa peroxisomal membrane protein, PMP70, before embedding for TEM as described [10]. In brief, a mouse eye sacrificed at 1.5 hours after light onset was fixed in 4% paraformaldehyde. Sections were cut using a cryostat and permeabilised using 0.02% saponin in 1% BSA in PBS for 30 mins. Antibody labelling was performed using an antibody against PMP70 (Novus Biologicals) in 1% BSA in PBS overnight at 4°C. Secondary Nanogold (Nanoprobes) in 1% BSA in PBS was applied to the sections for 2 hrs before re-fixing in 2% PFA, 2% glutaraldehyde in 0.1M cacodylate buffer for 1 hr. Gold enhancement solution (Nanoprobes) was applied to the sections following the manufacturers guidelines on ice at 4°C before preparing for TEM as described below.

### BSA-gold incubation

For the BSA incubation experiment, BSA was coupled to 10-nm colloidal gold particles as previously described [17]. Cells were incubated with BSA-gold in DMEM media for 2 hours (pulse) followed by DMEM only for 4 hours (chase). The sample was fixed in 2% paraformaldehyde/2% glutaraldehyde and prepared for TEM.

### Transmission electron microscopy sample preparation

Samples that had been fixed for TEM were incubated with aqueous 1.5% potassium ferricyanide, 1% osmium tetroxide for 1 hr in the dark at 4°C. Subsequently, the samples were dehydrated in an ethanol series (70%, 90% and 100%) before incubating in a propylene oxide and a mixture of propylene oxide and epon at 1:1. The samples were left in epon overnight at 65°C to generate sample blocks. Sections were cut using an ultramicrotome.

### Imaging

Images of TEM sections were acquired using a JEOL 1010 or JEOL 1400+ electron microscopes fitted with a Gatan Orius SC1000B charge-coupled device camera.

### Software development

**Fig 1:**
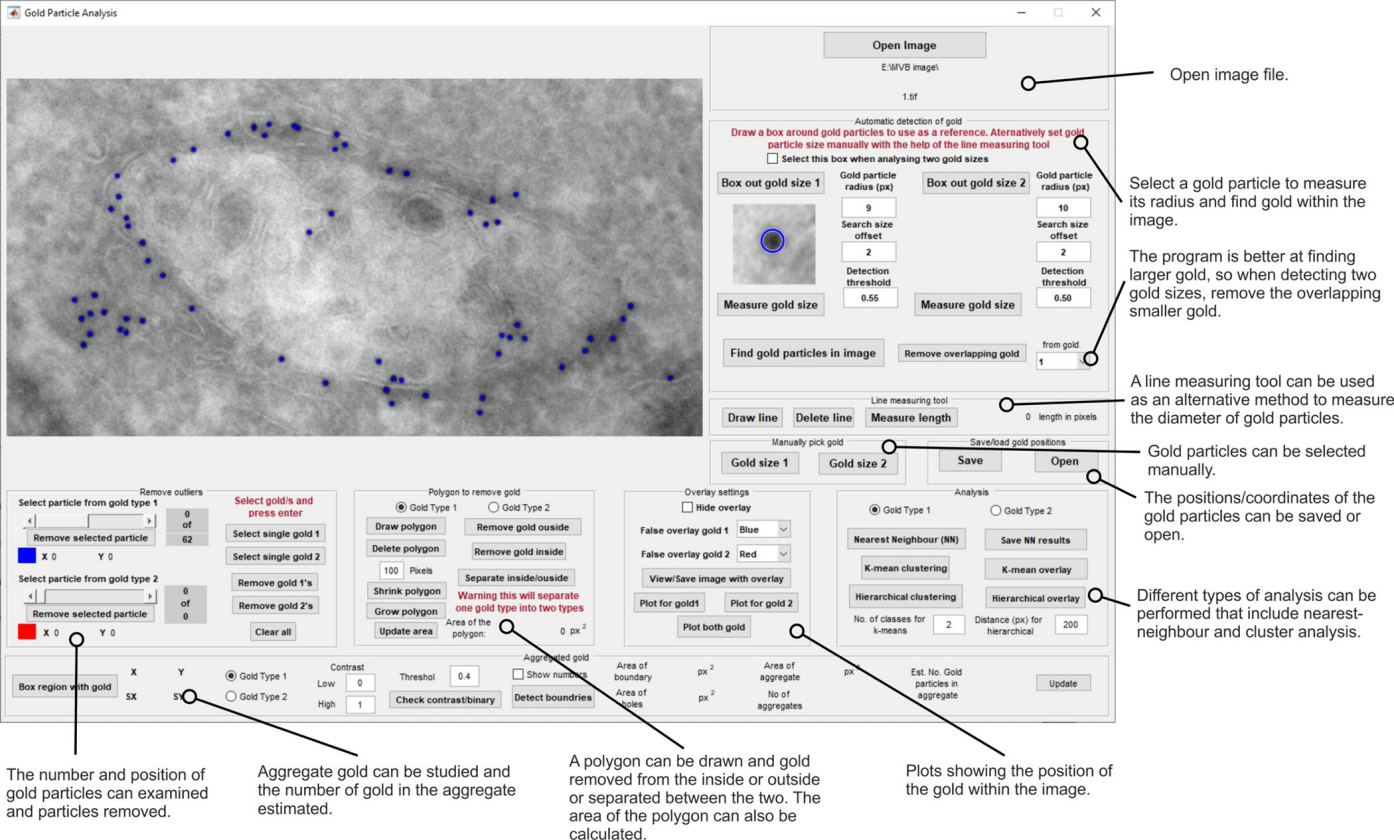
Gold Particle Analyser interface showing the different tools to assess gold particles in TEM images.

Gold Particle Analyser was developed using MATLAB 2020a (Mathworks). A range of features were included to allow detection and analysis of gold particles as shown in Fig 1. The software can be download from https://doi.org/10.5522/04/22005302

## Results

### Quantitation of immunogold particle distribution within endosomes after labelling of thawed cryosections

**Fig 2:**
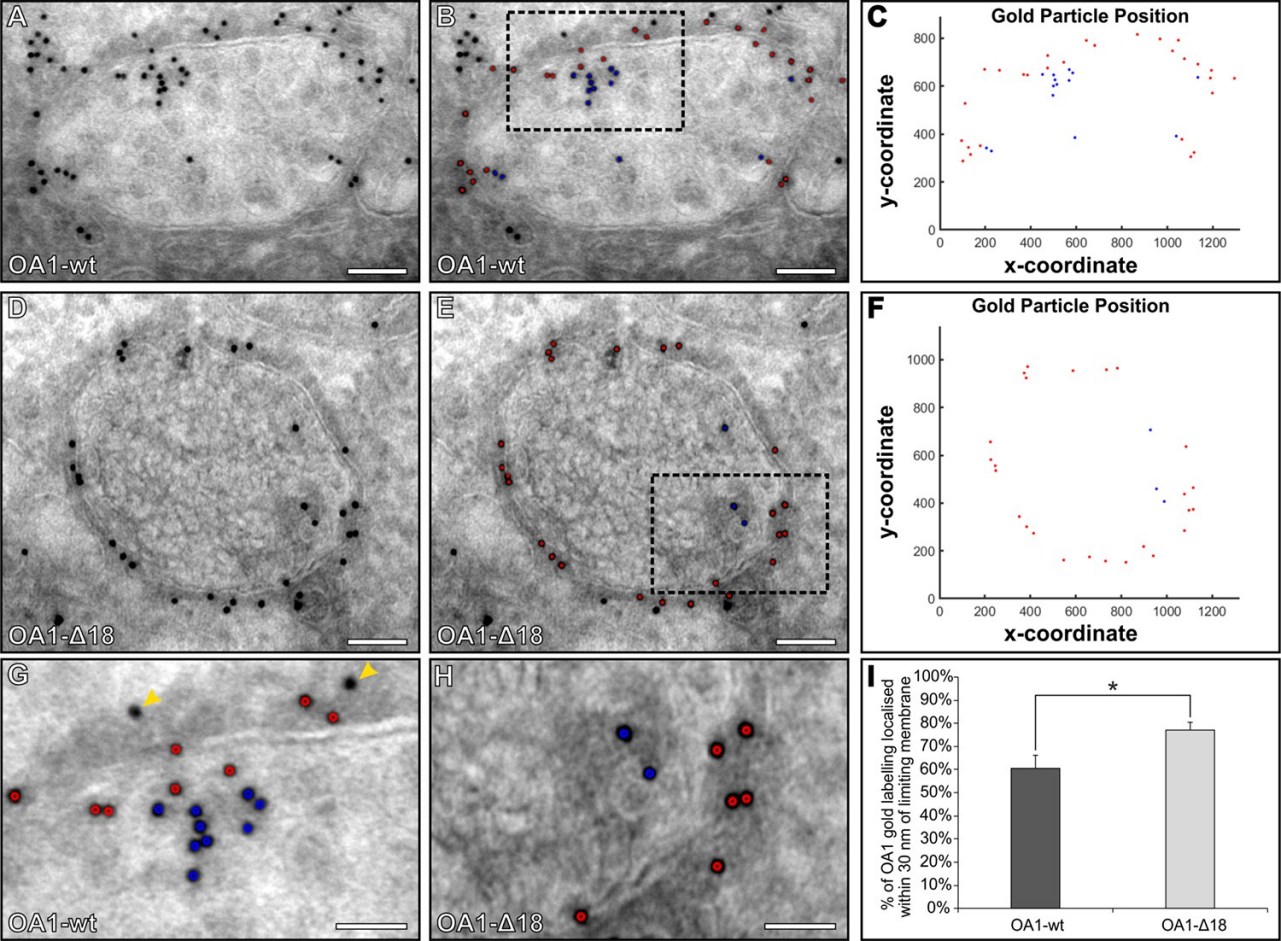
More OA1-Δ18 is localised at the limiting membrane of endosome compared to wild-type OA1 (OA1-wt). (A-B, D-E and G-H) Electron microscopy images of endosomes in HeLa cells expressing myc tagged OA1-wt or OA1-Δ18 labelled with an antibody against myc and 10 nm PAG. (B & E) Using Gold Particle Analyser, gold particles within 30 nm of the limiting membrane (overlaid in red) and those within the endosome more that 30nm away from the limiting membrane (overlaid in blue) were selected. To see how these selections were done refer to S1 Fig. (C & F) Plots showing location of the gold particles within the endosomes, gold particles within 30 nm are in red and those within the endosome in blue. (G & H) Zoomed in regions of the boxes shown in images (B & E). (G) The two gold particles indicated by the yellow arrowheads are on the outside of the endosome and are more than 30 nm away from the limiting membrane and are therefore not included in the analysis. (I) By taking the total number of gold particles at the limiting membrane and within the endosomes the % of OA1 at the limiting membrane was determined. A Student’s t-test determine a significant difference in the localisation of gold particles to the limiting membrane between OA1-wt and OA1-Δ18 * P ≤ 0.05 (n = 8 endosomes for each sample). Scale bars (A-B, D-E) 100nm and (G-H) 50nm.

Endosomal localisation of myc-tagged wild type (OA1-wt) or an OA1 mutant (OA1-Δ18) that shows reduced sorting onto the ILVs of endosomes, was investigated in TEM images of thawed cryo-sections using Gold Particle Analyser. Endosomes were identified by their ILV content and images acquired (Fig 2A and 2D). After automatically detecting gold particles within these images, the boundary drawing tool in Gold Particle Analyser allowed the separation between gold particles at the limiting membrane and within endosomes (see S1 Fig). To automatically detect the gold particles, drawing a polygon around the limiting membrane of an endosome and separating the internal and external gold in Gold Particle Analyser takes under 1 minute. As a bridging antibody (rabbit anti-mouse) was used in combination with a mouse anti-myc antibody, any gold within 30 nm either side of the limiting membrane signified potential protein localised to this membrane. Importantly, gold particles slightly more than 30nm from the limiting membrane, which would be difficult to exclude through manual counting without making time-consuming measurements, are automatically excluded (see Fig 2G). Gold at the limiting membrane is highlighted by red circles, whereas within endosomes they are blue circles as shown in Fig 2B, 2C, 2E–2H. The distribution of the gold can be visualised within x- y- coordinate plots that show a greater number of blue ILV associated gold in the OA1-wt compared to OA1-Δ18 (Fig 2C and 2F). Quantification of the number of gold particles within 30 nm of the limiting and within endosomes, showed more OA1-Δ18-myc localised to the limiting membrane (Fig 2I).

### Quantitative analysis of the number and distribution of rhodopsin immunogold particles to measure phagosome processing

**Fig 3:**
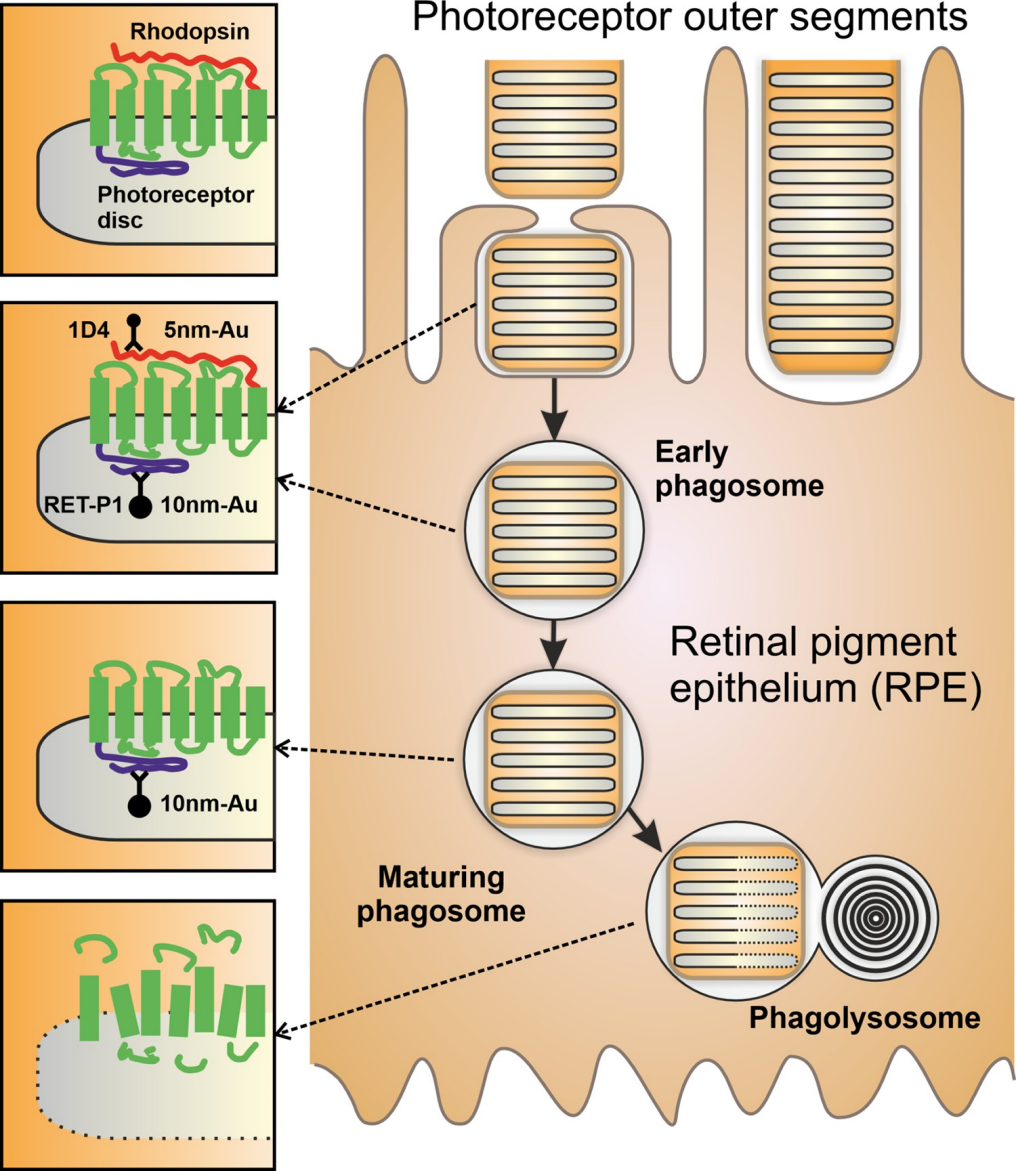
Diagram of photoreceptor outer segment (POS) phagocytosis by the retinal pigment epithelium (RPE). The distal tip of POS are phagocytosed daily by the opposing layer of RPE cells. Early phagosomes contain disc membranes that contain the same density of the light detecting rhodopsin protein as the POS themselves. As the phagosomes mature the C-terminal 1D4 epitope is lost before lysosomal fusion whilst degradation of the N-terminal RET-P1 epitope is only lost after phagosome:lysosome fusion.

**Fig 4:**
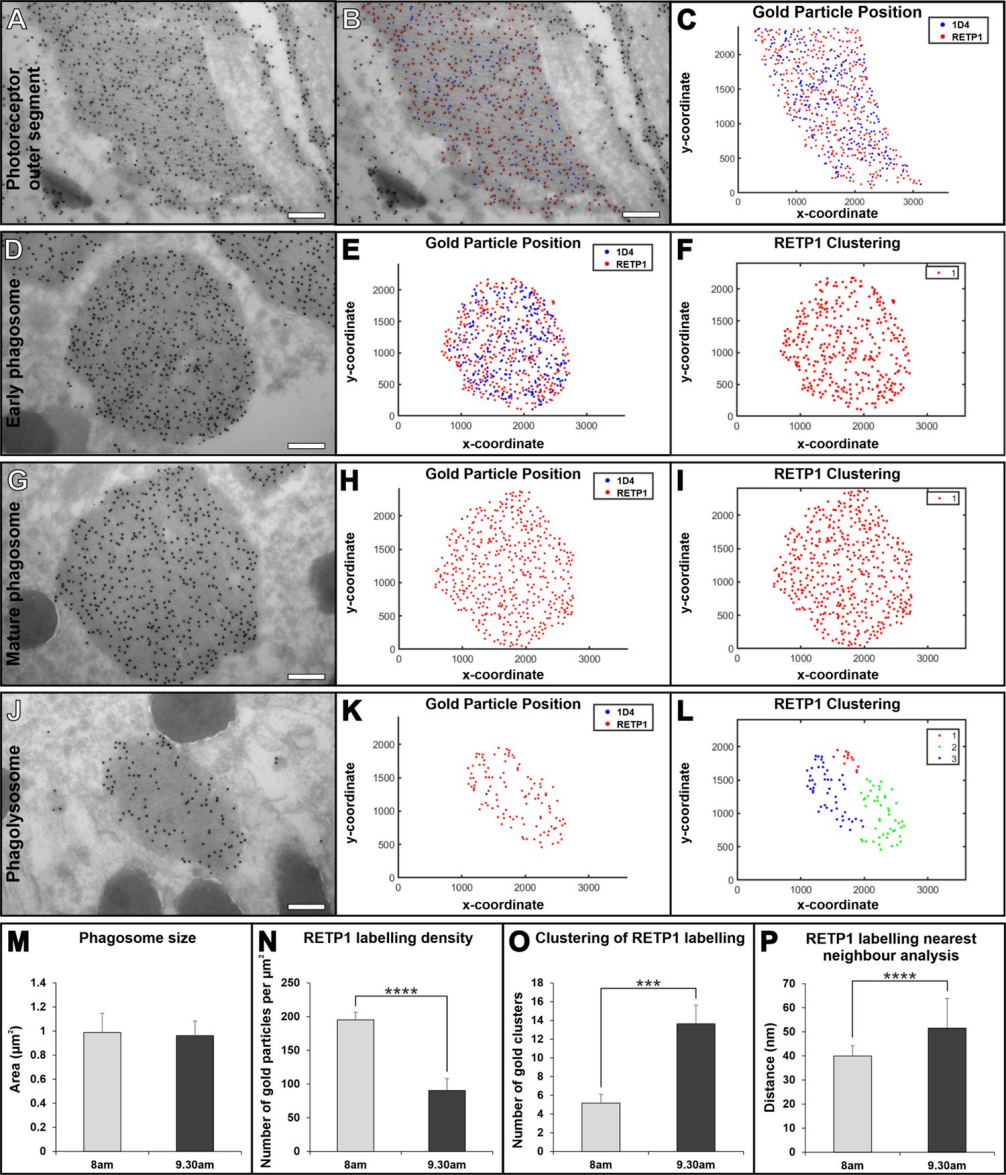
Mouse eyes examined at a later timepoint after light onset have more mature POS containing phagosomes within the RPE that have a greater loss of the RET-P1 rhodopsin epitope. (A—J) Representative electron microscopy images and analysis of non-phagocytosed proximal POS (A–C) and different stages of maturity of phagosome containing POS within the RPE that includes; early phagosomes (D–F), maturing phagosomes (G–I) and phagolysosomes (J–L). Images are from mouse retina prepared either 1 hour or 2.5 hours after light onset labelled with 1D4 (blue in overlays and graphs) and RET-P1 (red in overlays and graphs) in combination with 10nm and 15nm gold respectively. Proximal POS (A—C) have a similar rhodopsin labelling to early phagosomes containing POS (D–F). Mature phagosomes (G—I) have much less 1D4 epitope but little change in the RET-P1 as shown by the clustering (F, I). In phagolysosomes (J–L) there is less RET-PI indicated by the sparse gold labelling and multiple clusters compared to intermediate phagosomes (I—L). (F, I, L) Show hierarchical cluster analysis, where different colours represent different clusters, if a single colour is shown (F, I) there is a single cluster of gold particles detected. (M–P) Analysis of the measurements made by Gold Particle Analyser of images of phagocytosed POS in RPE at 1 hour and 2.5 hours after light onset. There is no difference in the size of the phagosomes between the 2 timepoints (M). At 2.5 hours after light onset there is less RET-P1 labelling within phagosomes (N) which is reflected in greater clustering (O) and larger nearest neighbour distance (P). Student’s t-test were used to determine a significance *** P ≤ 0.001 **** P ≤ 0.0001 (n = 15 images for each timepoint). Scale bars (A, B, D, G, J) 250nm.

The maturation of phagosomes containing photoreceptor outer segments was assessed within retinal pigment epithelial (RPE) cells of mouse retina isolated at 1 and 2.5 hours after light onset, timepoints previously shown to allow the transition from early to mature phagosomes and fusion with the lysosome to be followed (Fig 3). Antibodies against the rhodopsin C-terminus (1D4) and the N-terminus (RET-P1) were used in combination with two different sizes of PAG (10 nm for 1D4 highlighted in blue and 15 nm for RET-P1 highlighted in red in Fig 4). We previously showed that the combination of these antibodies allows the maturity of the phagosome to be determined; the 1D4 C-terminal epitope is lost before fusion with the lysosomes whilst the RET-P1 N-terminal epitope is only lost after lysosomal fusion (shown in Fig 3 and [13]). Early phagosomes have a similar density of labelling of both gold particles to the photoreceptor outer segments and cluster analysis reveals a single cluster of RET-P1 gold particles, indicating a relatively even distribution of the labelling within the phagosome (Fig 4A–4F). Mature phagosomes that have lost the 1D4 epitope but retain the RET-P1 epitope (Fig 4G and 4H) show a similar single cluster of RET-P1 gold particles (Fig 4F and 4I). After fusion with the lysosome, shown by reduced RET-P1 labelling, the phagolysosome typically contained 2 or more separated clusters of RET-P1 labelling (Fig 4J–4L). This clustering is likely due to non-homogeneous degradation of the RET-P1 epitope leading to groups of gold particles (Fig 4L). This could be a result of proteases better accessing some regions of the phagosome over others. Measurement of the phagosome size using the Gold Particle Analyser boundary drawing tool showed there was no significant difference between 1 and 2.5 hours after light onset (Fig 4M). However, consistent with phagosome maturation and phagosome:lysosome fusion the density of RETP1 labelling decreased while there was greater clustering and larger distance between gold particles between the earlier and later timepoint (Fig 4N–4P). From the images used in this analysis the accuracy of Gold Particle Analyser at detecting both sizes of gold was calculated to be 96.12% (N = 5964 particles detected). This included 3.21% of gold particles that were not detected and 0.69% picked up as the incorrect particle size.

### Detection and distribution of enhanced gold that labels peroxisomes following pre-embedding labelling

**Fig 5:**
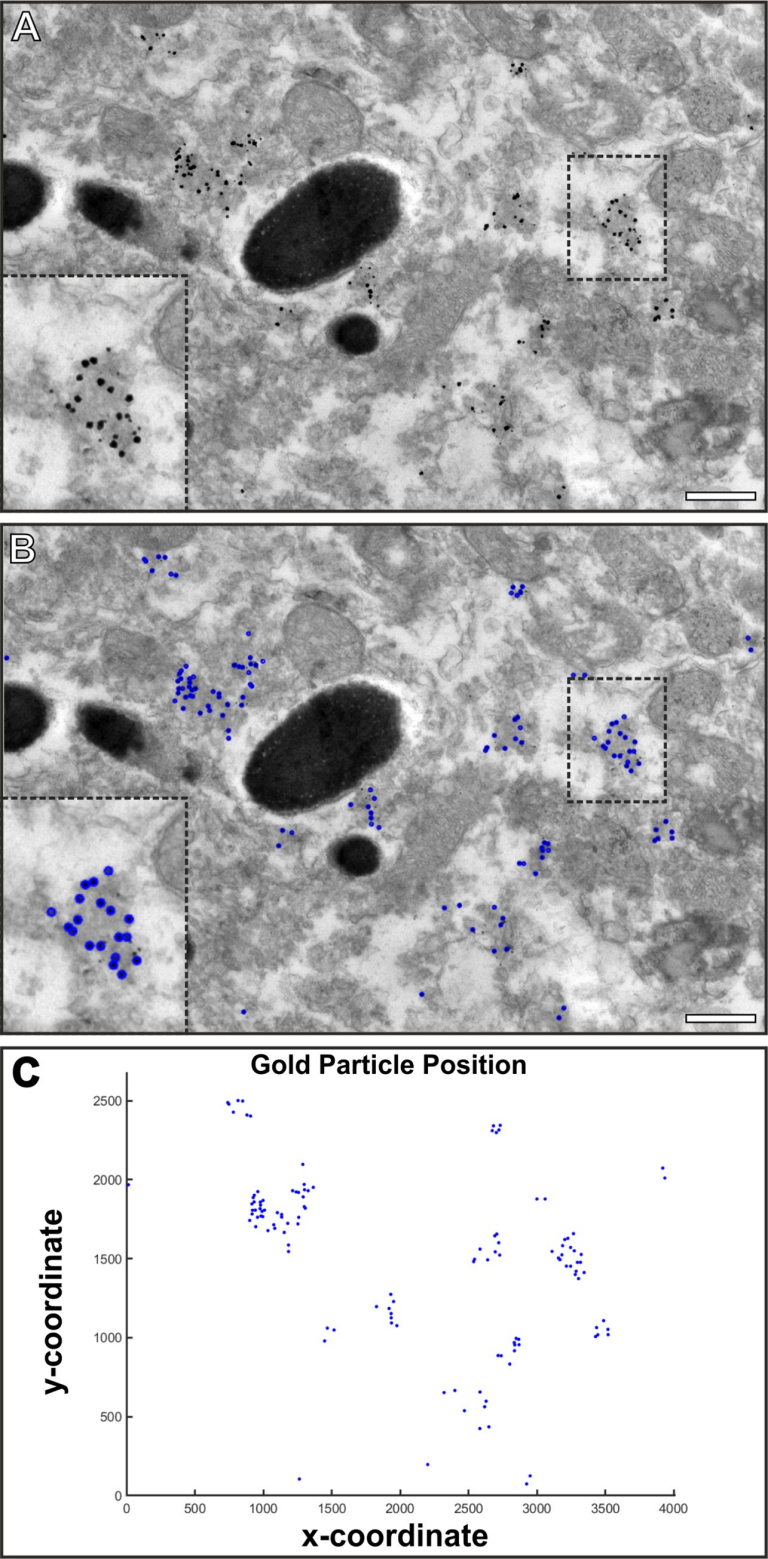
Gold Particle Analyser is able to detect irregular shaped and sized gold particles when pre-embedding labelling tissue using an antibody against PMP70. (A) TEM image of an RPE cell that has been immuno-gold labelled using an antibody against PMP70 and a secondary Fab-nanogold before enlarging the gold particles using gold enhancement. (B) The irregular shaped and size gold particles that are generated by the gold enhancement are detectable by Gold Particle Analyser and are highlighted by the blue circles. (C) A plot of the position of the gold particles within the images (blue points). Scale bars (A, B) 500 nm.

Peroxisomes were labelled within cryostat sections of mouse retina and images acquired of the RPE cell layer. Due to the pre-embedding methodology the gold enhancement results in irregular size and shaped gold particles. Gold Particle Analyser can be configured by adjusting the search size offset (large value for a greater range of gold sizes) and detection threshold (large value will detect more irregular shaped objects) to detect the gold automatically (Fig 5). In this case gold particle radius was set to 10, size offset was to 4 and detection threshold to 0.55. The smaller particles that had not enlarged (see panels Fig 5A & 5B) were excluded as these sometimes represent background/nonspecific labelling.

### Assessment of the size and number of gold particles within an aggregate

**Fig 6:**
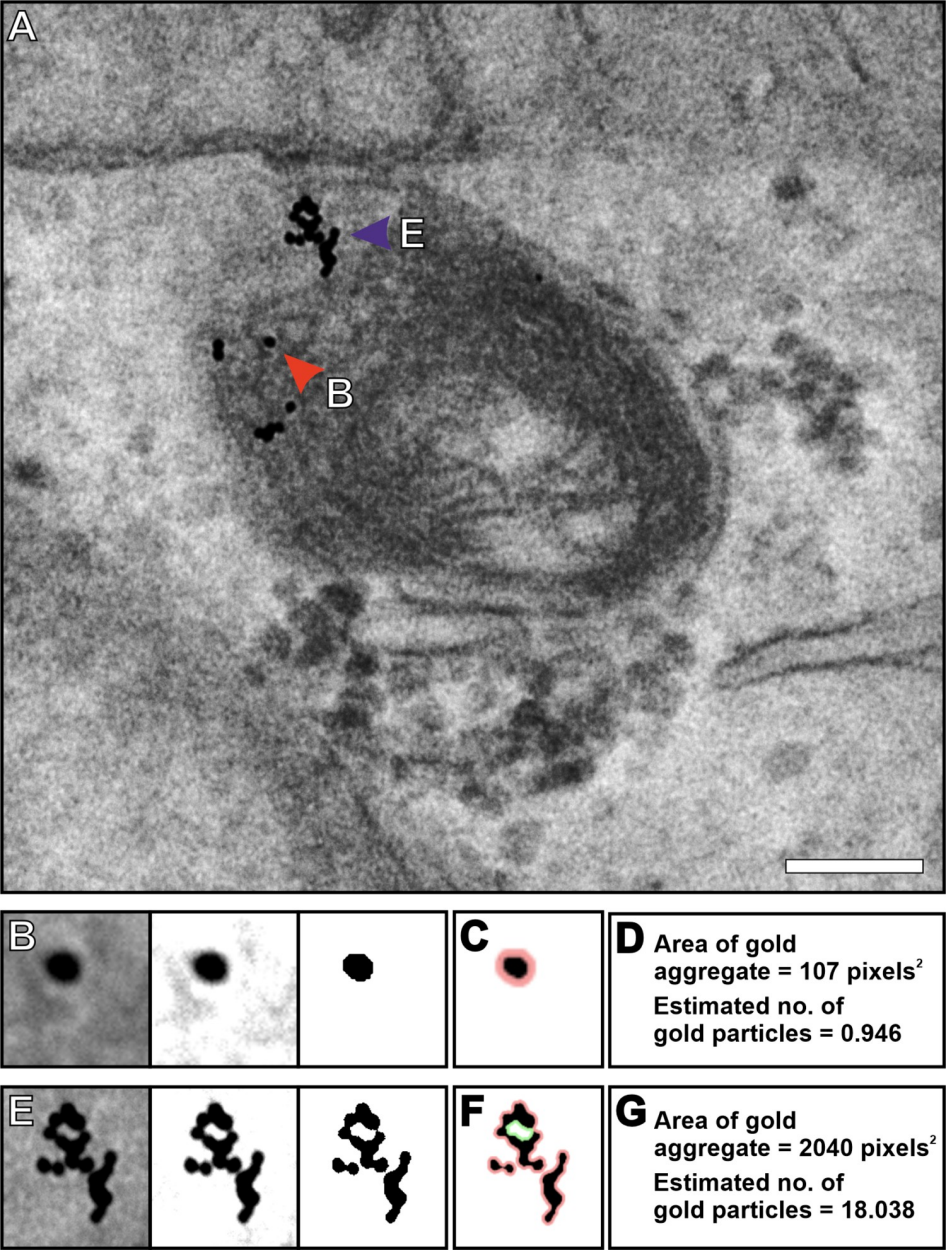
Estimation of the number of gold particles within an aggregate determine by Gold Particle Analyser. (A) Image of a lysosome within a HeLa cell incubated with BSA conjugated to 10 nm gold particles (BSA-gold). It is identified as a lysosome by aggregation of gold particles. (B) An image of a single gold particle selected in Gold Particle Analyser (left image) and the contrast modified (middle image) and converted to binary (right image) to allow detection of the perimeter as shown in (C) in pink. (D) This leads to the calculated area of the gold particle and an estimation of approximately one gold particle as expected. (E) An aggregate of gold particles (left image) that has been contrasted (middle image) and converted to binary (right image) before detecting the boundary (F). (F, G) The boundary around the selected gold particles (pink around the outside and green the boundary on the inside) leads to an estimation of 18.038 gold particles within the aggregate. Scale bars (A) 100nm.

An estimation of the extent of gold particle aggregation was determined in HeLa cells incubated with BSA conjugated to 10 nm colloidal gold. An endosome containing BSA-gold was determined to be fused to a lysosome by the presence of gold aggregation as a result of the acidic environment (Fig 6A). Gold Particle Analyser has a feature that detects the perimeter of single or multiple gold particles by removing the surrounding contrast before converting to binary format (Fig 6B, 6C, 6E and 6F). The program calculates the area of the gold particle or aggregate in pixels^2^ and uses this to determine the number of gold particles (Fig 6D and 6G). For this calculation to work, the program needs the radius of a single gold particle to be entered manually in pixels or by boxing out a single gold particle and using the automated measure gold size feature. Taking a single gold particle, the program estimated that it contained 0.946 gold particles (Fig 6D) and provided an estimation of 18.038 gold particles within an aggregate (Fig 6G). A further example of the analysis of clustered gold is shown in S2 Fig. These examples (Fig 6 and S2 Fig) show gold of a single size. When analysing samples containing varying gold sizes, the number of gold particles cannot be estimated but the area of the cluster can be calculated.

## Discussion

The introduction of gold particles to cells by endocytosis or immunogold labelling provides essential tools to look at protein localisation and the trafficking pathway at an ultrastructural level. To assist in the analysis of gold particles by TEM we have developed the program Gold Particle Analyser that includes a range of tools to automatically detect and assess gold particles. The program is able to assist in the analysis of gold within or on the membrane of organelles. By examining images of immunogold labelling in HeLa cells transfected with OA1-myc and OA1-Δ18-myc we were able to recapitulate previous findings there were determined by manual measurements [12]. More OA1-Δ18-myc is localised to the limiting membrane of endosomes than OA1-myc. The sorting of proteins from the limiting membrane to ILVs of endosomes is essential for normal cellular processes. It has a direct role in regulating signalling by removing the catalytic domain of receptors like EGFR from the cytoplasm, as well as targeting membrane proteins to the lysosome for degradation [18]. ILVs are also involved in melanosome biogenesis and are the precursors of exosomes released when multivesicular endosomes fuse with the cell surface [19]. Intensive study over the last two decades have resulted in the identification of the ESCRT machinery that regulates some but not all types of ILV formation [20]. How ESCRT-dependent and ESCRT-independent mechanisms are co-ordinated to form ILVs with different fates remains to be established, with studies hampered by the fact that ILV formation and cargo selection can only be directly studied by TEM. A streamlined unbiased method to measure sorting within MVBs using Gold Particle Analyser will facilitate studies of this fundamental biological process.

Gold Particle Analyser has been designed to detect up to two sizes of gold, allowing double immunogold labelling of two separate epitopes (within a single protein or two separate proteins). We challenged Gold Particle Analyser using cryo-immunoEM staining of two different rhodopsin epitopes on RPE cells that had recently phagocytosed photoreceptor outer segments. The huge density of rhodopsin staining means that the large and small gold particles are very tightly packed making manual counting challenging. Nevertheless we had previously shown that the rhodopsin C-terminus is lost prior to detectable delivery to lysosomes, whereas degradation of the N-terminus needs lysosomal enzymes including cathepsin D [13]. The results from Gold Particle Analyser agree with our previously published work [13]. At an early timepoint after light onset most phagosomes retained both rhodopsin epitopes, whilst at a later time there were more phagosomes that had lost the C-terminal epitope but retained the N-terminal epitope. Thus, the automated gold detection system within Gold Particle Analyser can rapidly and efficiently quantify the relative density of different sized gold particles even when densely packed. Additionally, at the later timepoint vacuoles with reduced N-terminal staining were present. This is difficult to assess by eye but the gold density, clustering and nearest neighbour analysis clearly showed a reduction in the density of rhodopsin N-terminal gold labelling, as well as providing quantitative evidence of spatial variation in the rate of rhodopsin degradation within the phagosome.

Some immunogold or gold incubation studies require examination of irregular size and shaped gold. Pre-embedding labelling involves enlarging nanogold (<1.5 nm) particles during the sample preparation process which tends to produce larger particles that are heterogeneous in shape and size. Examining mouse eye tissue that included pre-embedding labelling of the peroxisomal marker, PMP70, allowed us to determine if Gold Particle Analyser could automatically detect these particles. By changing parameters within the program for gold size range (size offset) and shape (detection threshold), it successfully allowed automated detection of enhanced gold particles. Gold that was not enlarged by the gold enhancement process was excluded as these are sometimes background/unspecific staining. If desired these can be detected by Gold Particle Analyser by increasing the size offset setting or using the two gold particle size setting.

Degradation of endocytosed proteins such as BSA bound to gold in the acidic environment of lysosome, results in gold aggregation [12]. This provides a useful technique to identify lysosomes and assess their activity. By TEM lysosomes can be morphologically heterogeneous in terms of their content of internal membranes, vesicles and electron density. Early endosomes, later endosomes and lysosomes can be filled with BSA-gold by varying the length of the incubation or including a chase to specifically load lysosomes. The presence and extent of BSA gold aggregation will indicate if the environment is acidic and degradative. The ability of Gold Particle Analyser to estimate the number of gold particles in aggregates, as shown here, could be useful for studies that involve manipulation or examination of lysosomal activity in disease models including age related macular degeneration, Stargardt’s early-onset retinal degeneration and Niemann-Pick disease type C [21–23]. Images generated by a TEM are a projection of the depth information of a section, making it difficult to access gold particles that are on top of each other. Therefore, the estimation of the number of gold particles has its limitations, but as aggregates form a 3D mass, this can still provide insight into changes in gold particle clustering under different conditions.

Gold Particle Analyser includes additional features compared with existing automated gold analysis programmes [3–5]. These include features to help analyse clustered or irregularly sized enhanced gold particles. Previous approaches have been developed to measure the distance between gold particles and cellular membranes [3]. Our feature to allow separation of gold either side of membranes, allows for a different type of analysis that can be particularly useful in the membrane trafficking field. Additional features of Gold Particle Analyser include cluster and nearest neighbour analysis. A limitation of Gold Particle Analyser is that it has been designed to analyse TEM images of organelles rather than whole cells, in contrast to programs such as Gold finder that are able to detect gold within montaged images of whole cells [5]. Gold Particle Analyser also does not automatically distinguish between specific and non-specific labelling. When performing immuno-gold labelling of thawed cryo-sections, it is known that some antibodies result in non-specific labelling of organelles such as mitochondria and nuclei [1]. If the ratio of labelling that is considered non-specific and specific, needs to be calculated within an image the non-specific labelling can be removed using the polygon tool, before doing the opposite and removing specific labelling, and in both cases the total number of gold particles recorded to calculate the ratio.

In summary, Gold Particle Analyser is a novel free program that can assist in studies globally that involve immunogold labelling and gold probes. This includes research to better understand cellular processing including trafficking, organelle structure and function and disease models. Gold Particle Analyser has been designed to be easy to use and to provide tools to analyse gold particles that cannot be achieved manually or within any other single program that is available.

## Supporting information

S1 Fig Step-by-step guide to separate gold particle at the limiting membrane and within endosomes using Gold Particle Analyser. Scale bar 50nm.
(PDF)

S2 Fig A further example showing the estimation of the number of gold particles within an aggregate determine. (A) Image of an endolysosome within a HeLa cell incubated with BSA bound to 10 nm gold particles (BSA-gold). (B) An image of single gold particle selected from (A) in Gold Particle Analyser (left image) and the contrast modified (middle image) and converted to binary (right image) to allow detection of the perimeter as shown in (C) in pink. (D) This leads to the calculated area of the single gold particle as approximately one gold particle, which is expected based on the scale information of the image used. (E) An aggregate of gold particles (left image) that has been contrasted (middle image) and converted to binary (right image) before detecting the boundary (F). (G) The boundary around the selected gold particles leads to an estimation of 14.35 gold particles within the aggregate. When counting the particles by eye, there appears to be around 14 gold particles visible within this cluster. Scale bars (A) 50nm (C, F) 10nm.
(PDF)
