# Supplementary material for: Gold Particle Analyser: Detection and quantitative assessment of electron microscopy gold probes
Source: PLoS One. 2023 Jul 28;18(7):e0288811. doi: 10.1371/journal.pone.0288811 (PMC10381077; doi:10.1371/journal.pone.0288811)
Supplement: S1 Fig — Scale bar 50nm. (PDF) [file pone.0288811.s001.pdf]

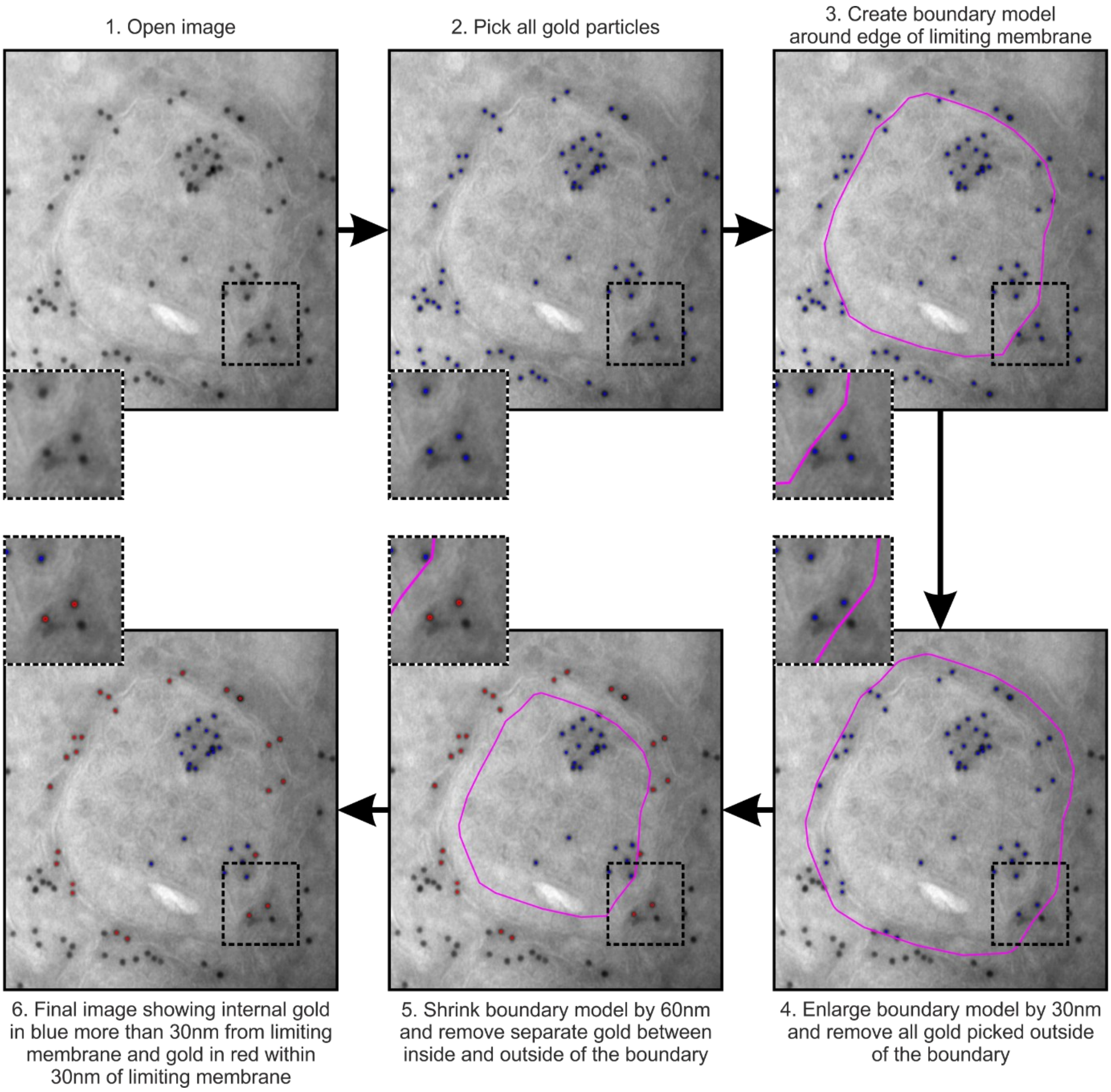

Fig S1. Step-by-step guide to separate gold particle at the limiting membrane and within endosomes using Gold Particle Analyser. Scale bar 50nm.
