# Supplementary material for: Gold Particle Analyser: Detection and quantitative assessment of electron microscopy gold probes
Source: PLoS One. 2023 Jul 28;18(7):e0288811. doi: 10.1371/journal.pone.0288811 (PMC10381077; doi:10.1371/journal.pone.0288811)
Supplement: S2 Fig — (A) Image of an endolysosome within a HeLa cell incubated with BSA bound to 10 nm gold particles (BSA-gold). (B) An image of single gold particle selected from (A) in Gold Particle Analyser (left image) and the contrast modified (middle image) and converted to binary (right image) to allow detection of the perimeter as shown in (C) in pink. (D) This leads to the calculated area of the single gold particle as approximately one gold particle, which is expected based on the scale information of the image used. (E) An aggregate of gold particles (left image) that has been contrasted (middle image) and converted to binary (right image) before detecting the boundary (F). (G) The boundary around the selected gold particles leads to an estimation of 14.35 gold particles within the aggregate. When counting the particles by eye, there appears to be around 14 gold particles visible within this cluster. Scale bars (A) 50nm (C, F) 10nm. (PDF) [file pone.0288811.s002.pdf]

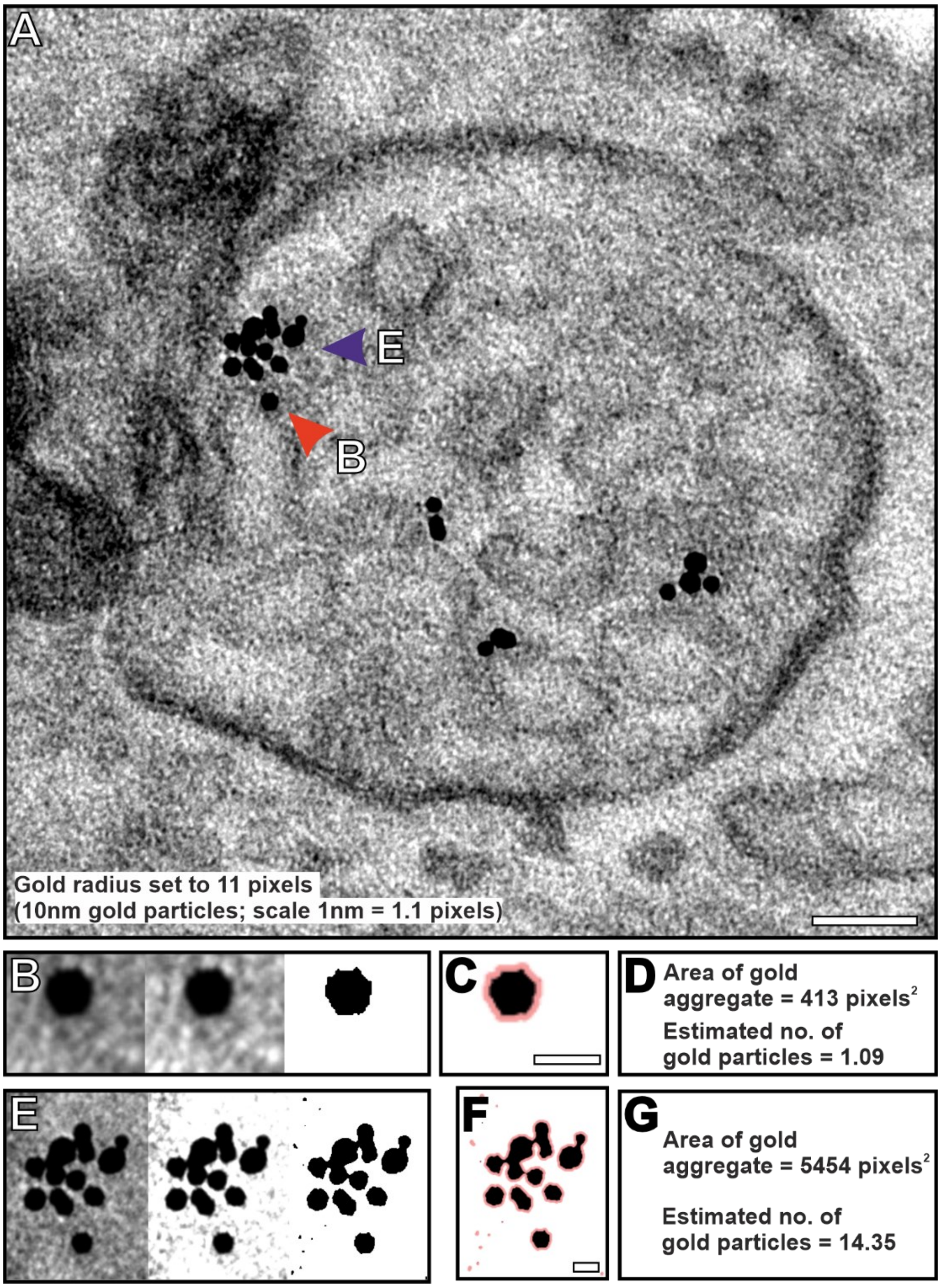

Fig S2. A further example showing the estimation of the number of gold particles within an aggregate determine. (A) Image of an endolysosome within a HeLa cell incubated with BSA bound to 10 nm gold particles (BSA-gold). (B) An image of single gold particle selected from (A) in Gold Particle Analyser (left image) and the contrast modified (middle image) and converted to binary (right image) to allow detection of the perimeter as shown in (C) in pink. (D) This leads to the calculated area of the single gold particle as approximately one gold particle, which is expected based on the scale information of the image used. (E) An aggregate of gold particles (left image) that has been contrasted (middle image) and converted to binary (right image) before detecting the boundary (F). (G) The boundary around the selected gold particles leads to an estimation of 14.35 gold particles within the aggregate. When counting the particles by eye, there appears to be around 14 gold particles visible within this cluster. Scale bars (A) 50nm (C, F) 10nm.
